# Optimizing Care Across the Continuum for Older Adults with Lung Cancer: A Review

**DOI:** 10.3390/cancers16223800

**Published:** 2024-11-12

**Authors:** Leah Thompson, Caterina Florissi, Jaewon Yoon, Anupama Singh, Anurag Saraf

**Affiliations:** 1 Department of Radiation Oncology, Brigham and Women’s Hospital, Boston, MA 02115, USA; 2 Harvard Medical School, Boston, MA 02115, USA; caterina_florissi@hms.harvard.edu (C.F.);; 3 Department of Surgery, University of Massachusetts Chan Medical School, Worcester, MA 01655, USA; anupamasingh0312@gmail.com

**Keywords:** older adult, geriatric oncology, comprehensive geriatric assessment, lung cancer

## Abstract

Older adults with lung cancer experience inferior clinical outcomes compared to their younger counterparts. This review provides the scaffolding to address these disparities by delineating (1) the distinct and varied care needs of older adults with lung malignancies, (2) evidence-based measures for identifying subgroups within this population meriting tailored approaches to care, (3) age-specific considerations for the selection of cancer-directed therapy, and (4) opportunities for future work to enhance clinical outcomes and care delivery.

## Simple Summary

Lung cancer is the leading cause of cancer-related mortality among older adults, but older adults experience age-related disparities in treatment outcomes, clinical trial representation, and guideline-concordant care. This review addresses critical factors contributing to these disparities, outlining age-specific considerations to optimize management of medical and supportive care needs and cancer-directed therapy selection. Key recommendations include comprehensive geriatric assessment, individualized treatment planning, and improved resource allocation to ensure older adults receive equitable and effective care.

## 1. Introduction

Cancer disproportionately impacts older adults, a population with a heterogeneous constellation of medical, psychosocial, and supportive care needs [1,2,3,4,5,6,7,8,9,10]. As the global population ages, absolute disease burden in those over 65 is only expected to increase [1]. Yet despite these trends, age-related disparities in clinical outcomes persist. As compared to their younger counterparts, older adults with cancer experience worse survival outcomes, higher rates of treatment-related toxicity, greater variation from guideline-concordant care, and unequal clinical trial enrollment [11,12,13,14].

Paralleling findings across cancer types, age-specific differences in disease burden and outcomes endure in thoracic oncology. Though therapeutic advances over the last decade have improved prognosis for select subgroups, lung cancer remains the leading cause of cancer-related death in the United States, and older adults have up to a two–three-fold increased risk of dying from their disease [15,16]. Beyond survival, older adults with lung cancer, similar to the general geriatric oncology population, are under-represented in thoracic clinical trials and have a lower likelihood of receiving timely guideline-concordant therapy [17,18,19,20,21,22].

Parsing the reasons for these observed disparities is complex. At the patient-level, differences in medical and supportive care needs, frailty, functional status, treatment toleration, and social supports may be contributors [8,23,24,25,26,27,28,29,30,31,32,33,34,35,36]. At a systems-level, the existing limitations in resource allocation, geriatric assessment utilization, and age-inclusive research may all be relevant [3,19,21,37,38,39,40,41,42]. Older adults with lung cancer constitute a disparate population with varied needs and care environments, and in specific circumstances, one of more of these factors may be more significant [1,2,3,4,5,6,7,8,9,10,23,24,25,26,27,28,29,30,31,32,33,34,35].

However, though future investigations are needed to untangle the interplay between these factors in different contexts, practical optimization of care for older adults with lung cancer remains possible in the present. An array of age-specific resources and guidelines already exist with demonstrated value across multiple facets of thoracic oncology care, although usage remains variable [8,17,18,24,37,38,39,40,41,42]. To support clinicians caring for older adults with lung cancer, outlining the features of this population, validated tools for care optimization, and best-practice recommendations are valuable. Further, a number of brief screening tools have been validated to assess specific domains for older patients with NSCLC (Table 1).

This review provides the scaffolding to support these aims by delineating the following: (1) the distinct and varied care needs of older adults, (2) evidence-based measures for identifying subgroups within this population meriting different approaches to care, (3) age-specific considerations for selection of cancer-directed therapy, and (4) opportunities for future work to enhance clinical outcomes and care delivery. 

## 2. Medical Needs

Older adults with lung cancer have a heterogeneous array of medical needs which deserve consideration across the care continuum. Issues specific to older adults in this domain include multimorbidity, and polypharmacy, as well as frailty and other geriatric syndromes. Each of these issues is discussed further below. 

### 2.1. Multimorbidity and Polypharmacy 

Multimorbidity and polypharmacy are central considerations for providers managing older adults with lung cancer. The comorbidity burden increases with age, and older adults with lung cancer have, on average, a median of two other chronic conditions at the time of cancer diagnosis [79,80]. Multimorbidity matters because it may influence the time to diagnosis, treatment experiences, and survival [25,43,79,81,82,83,84]. Regarding treatment, both older adults and those with a greater comorbidity burden independently have a lower likelihood of receiving active therapy, suggesting potential compounding of these challenges in multimorbid geriatric populations [22,28]. Specific comorbidities may also constrain therapeutic options and increase the risk of select treatment-related toxicities (i.e., treatment-related pneumonitis in a patient with pre-existing chronic obstructive pulmonary disease). These challenges arise particularly commonly in thoracic oncology given the frequent co-occurrence of smoking-related cardiopulmonary comorbidities and lung cancer [80,82,83].

Beyond the direct effects of comorbidity burden on therapy selection and toxicity, older adults with multimorbidity may also experience difficulties related to polypharmacy, potentially inappropriate medications, and drug interactions [29,30,85]. In the general elderly population, the prevalence of polypharmacy, defined as the concurrent use of five or more drugs, remains high, ranging from 22.8 to 44% [29,86,87,88]. Similarly, in older adults with lung cancer, a recent systematic review and meta-analysis demonstrated a pooled prevalence of polypharmacy of 38% [30]. Though in some circumstances the use of numerous concurrent medications may be appropriate, presence of polypharmacy in geriatric oncology populations can be associated with an array of adverse outcomes including: functional impairment, frailty, falls, delirium, cognitive impairment, adverse drug reactions, drug–drug interactions, cancer-related treatment toxicity, hospitalization, and inferior overall survival [29,30,36,89,90,91,92,93,94,95,96,97]. Polypharmacy is also associated with other suboptimal medication-related practices which are independently associated with poor prognosis, including the usage of potentially inappropriate medications (PIMs), generally defined as medications that have an unfavorable benefit–risk ratio in older adults [85,98,99]. In older adults with lung cancer, PIM prescribing is common, impacting 28–43% of patients, and is associated with all-cause mortality [30]. 

Collectively, these findings underscore the importance of assessing for multimorbidity, optimizing comorbidities, collaborating with other specialists, and reducing inappropriate medication usage and polypharmacy where feasible. Ideally, these interventions would occur within the context of a larger comprehensive geriatric assessment (CGA). However, though optimal mechanisms for comprehensive assessment in resource-rich settings will be discussed in subsequent sections, it is worth noting that targeted screening for polypharmacy and PIMs can be quickly performed by any provider simply by counting medications prescribed and referencing one of several validated tools for PIM identification (Table 1) [50,51,52]. Similarly, screening for severe comorbidities and coordinating care with relevant specialists can readily be accomplished by clinicians within the confines of a clinical visit [43,44,45,46,47,48]. Consideration of even these types of narrow interventions has the potential to enhance the care of older adults with lung cancer.

### 2.2. Frailty and Geriatric Syndromes 

As aging occurs, all individuals are at an increased risk of geriatric syndromes: clinical conditions arising from accrued impairments in multiple systems coupled with individual inability to compensate for these derangements [24,100]. Unsurprisingly, older adults with cancer experience these syndromes at higher rates than their aging counterparts in the general population due to the coupled stressors of disease burden and cancer-directed therapy [101]. Common geriatric syndromes in older adults with cancer include frailty, falls, cognitive impairment, delirium, incontinence, and pressure ulcers [24,100]. These types of syndromes pose management challenges in thoracic oncology because they defy conventional organ-based categorizations, requiring a systems-based approach. They are relevant to the care of older adults given their associations with quality of life, functional impairment, hospitalization, success of cancer-directed treatments, and risk of death [24,101,102,103].

Of the array of geriatric syndromes, the most well studied in older adults with lung cancer is frailty. Frailty definitions vary, with different definitions conceptualizing it as physical disability, limitations in basic or instrumental daily activities (ADLs/iADLs), or simply as heightened vulnerability to adverse outcomes [104]. However, two general paradigms exist, with most models following one or the other approach. The first paradigm is the phenotype model of frailty, which incorporates five criteria: grip weakness, self-reported exhaustion, slow gait speed, low physical activity, and unintentional weight loss. In this model, patients are defined as frail if they meet three or more of these criteria [105]. The second paradigm is the deficit accumulation model of frailty, which counts the total number of health deficits an individual has (symptoms, disabilities, comorbidities, lab derangements, etc.). In this model, frailty is conceptualized along a spectrum, with higher scores indicating greater vulnerability [106]. Across models, effective assessments of frailty typically encompass some measure of the patients’ symptoms, functional impairment (physical, cognitive, and social), and body composition changes [55,107,108,109]. An array of specific screening instruments exist for usage in older adults with lung cancer and other neoplasms [53,54,55,56]. Importantly, as highlighted in a recent large meta-analysis of 2359 lung cancer patients, even across screening tools, the presence of frailty by any definition may be associated with higher overall mortality and therapeutic toxicity (HR 1.20, 95% CI 1.05–1.38; OR 1.72, 95% CI 1.18–2.51, respectively) [110]. Frailty has also been reliably shown to be associated with other meaningful measures of medical status, including body composition changes, symptom burden, and functional decline [108,111,112,113]. Irrespective of the specific tool used, these findings underscore the importance of identifying factors contributing to frailty in older adults with lung cancer and using this information to guide care decisions and toxicity management. 

As emphasized elsewhere, the evaluation of any single parameter of medical status, whether multimorbidity, polypharmacy, or frailty, should ideally occur within the framework of a CGA. However, for frailty, similar to other measures, it is again important to emphasize that even if a complete CGA cannot be performed on every older adult due to resource constraints, basic relevant information can still be collected. More specifically, weight changes, ability to perform iADLs/ADLs, and recent falls are datapoints related to frailty that can be readily captured in any clinical setting and should be obtained for every older adult with lung cancer. Importantly, there are also brief 8–13 question validated survey tools which can be used to identify vulnerable subgroups who would most benefit from a CGA (i.e., Vulnerable Elders Survey-13, G-8 Geriatric Screening Tool) [64,77,78]. Physicians should be emboldened to incorporate whatever metrics are feasible, within practice constraints, to capture facets of frailty in clinical care and identify high-risk individuals (Table 1). 

### 2.3. Supportive Care Needs 

Alongside medical needs, older adults with lung cancer also have a unique constellation of supportive care needs. Relevant domains include treatment-related side effects, socioeconomic supports and burdens, and advanced care planning. Each of these issues is discussed further below.

#### 2.3.1. Treatment-Related Side Effects 

Across patient groups, lung cancer is associated with substantial longitudinal symptom burden [114]. Interestingly, emerging evidence suggests that older adults with cancer may experience different patterns of physical and psychologic symptoms than their younger counterparts during treatment [5,7,111,115]. More specifically, though data quality is poor, investigations encompassing varied cancers generally suggest that older adults may experience a different intensity of specific symptoms (i.e., appetite change, psychological distress), and an overall larger and more varied array of physical and psychological concerns than their younger counterparts [6,7,111,115,116,117]. Treatment-related changes in weight, appetite, and nutrition may be of particular concern given the high rates of cachexia observed in geriatric oncology populations [118].

Older adults are also more likely to be undertreated for specific symptoms, such as pain [119,120,121]. These findings underscore the importance of monitoring for the full array of potential symptoms older adults may experience during treatment and promptly addressing concerns where present. 

Interestingly, for specific symptom clusters such as mood symptoms, data also suggest greater individual heterogeneity in symptom presentation among older adults, which can complicate identification [122,123,124]. Pseudodementia, a neuropsychiatric condition manifesting as neurocognitive and functional impairment mimicking neurodegenerative disease, is a well-documented phenomenon in older adults [123]. However, cognitive symptoms such as difficulty concentrating or forgetfulness may also simply reflect aging and not an underlying mood disorder [122]. In older adults with cancer, identifying clinical depression and anxiety is particularly challenging given the range of somatic symptoms associated with underlying disease and treatment, some of which may mimic psychological disorders [122]. These findings underscore the importance of evaluating treatment-related symptoms within a broader context rather than leaping to pathologize them. Report of specific symptoms during treatment should catalyze a broader dialogue about overall mood, coping strategies, and adequacy of existing support systems. In circumstances where a clinical mood disorder or substantial psychological distress is identified, providers should consider integrated multimodal approaches incorporating evidence-based psychosocial interventions, behavioral techniques, and pharmacotherapy where appropriate [125].

Beyond divergent patterns of symptoms, older adults with lung cancer may also experience different consequences of treatment-related symptoms on their daily functioning. For example, in one large study of 903 patients with solid tumors receiving therapy, 90% of older adults reported treatment-related symptoms interfering with quality of life (QOL) or daily functioning, and older adults had greater symptom-related distress than their younger counterparts [111]. Other studies in older adults with cancer have demonstrated similar relationships, irrespective of cancer type, disease stage, or number of comorbidities [126]. Mirroring these findings, in lung cancer-specific geriatric cohorts, studies have also shown consistent relationships between symptom burden, QOL, and functional impairment [127,128]. Treatment-related functional decline is of particular concern in older adults with lung cancer, particularly those with a pre-existing disability, because it may influence their ability to live independently and complete meaningful and necessary daily activities. Function and quality of life (QOL) changes arising from symptoms are also particularly relevant given the importance that older adults place on these factors. In one large study of 226 patients with limited life expectancy due to cancer or cardiopulmonary disease, over 70% reported they would not want a treatment resulting in functional impairment even if it improved survival [129]. These findings highlight the importance of identifying function and QOL priorities to support informed decision-making throughout the treatment process. Where feasible, clinicians should assess patient-reported symptoms, QOL, and frailty using available validated tools (Table 1) [55,57,59,60,61,62,63,64,78,130,131,132,133,134,135,136,137].

#### 2.3.2. Socioeconomic Supports and Burdens 

Cancer care occurs within a patient’s socioeconomic context. Economic consequences arising from cancer diagnosis and treatment, often termed “financial toxicity” (FT), are an important but often overlooked component of older adults’ experiences with cancer [138,139,140]. Approximately one-fifth of elderly patients with cancer experience treatment-related financial distress, and those endorsing financial strain also commonly report reduced QOL and higher rates of mood symptoms [139,140]. The best approach to screening for and mitigating these stressors remains unclear, with varied approaches in current use and no national consensus guidelines. Regarding screening, several available questionnaires exist that capture elements of financial toxicity, but further validation across contexts will be valuable [65,66,138,141,142]. Similarly, regarding mitigation, emerging evidence suggests that embedded financial navigation services may reduce out-of-pocket spending for patients. These services can generally assist with health insurance optimization, health literacy education, and referral to social support services. However, whether these interventions meaningfully reduce cumulative financial burden remains unknown [143]. To better address these factors shaping patient experiences, further investigation is needed to define best practices. 

Alongside the financial context, social support, defined as the constellation of individuals available to provide psychological, physical, and financial help to a patient, also plays a vital role in oncologic outcomes [144]. More specifically, existing evidence suggests support is closely associated with physical health, psychological adjustment, hospital readmission, and overall survival [145,146,147,148,149,150,151,152]. As aging occurs, social networks may be narrowed by death and illness, while concurrently, medical and supportive care needs may increase. In one recent survey of 1460 older adults with cancer, two-thirds reported at least one unmet social support need, underscoring the scope of the challenge [153].

Beyond the impact of age on the experience of patients, the caregivers of older adults may also experience physical, emotional, and financial stressors during their loved one’s cancer treatment. These caregivers are typically older adults themselves and may have similar vulnerabilities as their patient counterparts, with research suggesting at least a third have pre-existing fair to poor health and/or a serious health condition [154,155]. Seventy-five percent of caregivers report experiencing at least some degree of caregiver burden as the result of their loved one’s diagnosis, and up to one-fifth may experience a decline in QOL as the result of caregiving responsibilities [156,157]. This burden is heightened when patients are highly symptomatic and have reduced functional status, and concerningly, is associated with increased risk of mortality [158,159]. These findings emphasize the importance of a timely CGA, which will be discussed in subsequent sections, but can help identify individual patient and caregiver needs and mobilize appropriate resources. However, even in settings where comprehensive assessment is not possible for every patient, every clinician should inquire about capacity to perform basic and instrumental activities of daily living and existing social supports. An array of brief patient-and caregiver-facing validated screening tools that can be incorporated into clinic also exist, which can readily be used to identify patients at a particularly high risk for poor outcomes (Table 1) [67,68,69,70,71,160,161,162,163].

#### 2.3.3. Advanced Care Planning

Beyond attending to treatment-related and socioeconomic needs, engaging older adults with lung cancer in advanced care planning (ACP) is also of pivotal importance. ACP is the serial process through which patients delineate their goals, values, and preferences for medical care, and may include the exploration of care goals with clinicians and chosen community, completion of an advanced directive, and/or designation of a healthcare proxy (HCP) [164]. This process is the cornerstone of high-quality care in older adults with cancer, and is associated with more goal-concordant care delivery, increased caregiver and patient satisfaction, and reduced rates of high-intensity end-of-life care [72,165,166,167,168,169]. However, despite growing evidence suggesting most older adults with cancer want to participate in ACP early and iteratively, rates of engagement remain low [170,171]. Estimates vary, but in a recent large study of cancer patients aged 55 and older and their families, 65% reported discussing end-of-life care in any context, 61.9% had designated a medical decision-maker, and only 54.1% had advanced directives [172]. Alongside observed limitations in ACP, prior work has suggested that over half of older adults with cancer and their caregivers have prognostic perceptions diverging from their providers [9,173,174,175]. These findings highlight ongoing communication challenges meriting further interrogation in lung-specific populations. 

Meaningful advanced care planning is supported by a shared understanding of age-associated vulnerability, cancer prognosis, life expectancy, therapeutic tradeoffs, values, goals, and preferences [9]. Age may be a relevant consideration in these discussions as it can influence patient perspectives on tradeoffs between quality of life, function, and life-prolonging treatment [129,176,177,178,179]. Age may also influence preferences for mode of information delivery and mechanisms for coping with illness [6,177,178]. However, importantly, age is one factor of many. Older adults possess a varied suite of medical, supportive, and psychosocial needs, and identification of age group does not obviate individualized decision-making [1,2,3,4,5,6,7,8,9,10]. In older adults, as in younger patients, ACP should begin early and occur frequently. A variety of validated tools exist which may support clinicians engaging older adults with lung cancer on this important domain (Table 1) [72,73,74,75,76]. Other mechanisms for enhancing care-related communication, including timely palliative care engagement, have also been shown to increase ACP rates and enhance prognostic awareness across age groups [180,181,182,183]. As will be discussed further in subsequent sections, in older adults, these discussions are optimally informed by a preceding CGA [9]. 

## 3. Comprehensive Geriatric Assessment

The CGA begins with a multifaceted interdisciplinary evaluation which captures the medical, psychosocial, and functional needs of an older adult. The evaluation component of CGA leverages validated tools which assess function and mobility, medical comorbidities and polypharmacy, cognition, psychological state, and nutrition, as well as social functioning and supports [184,185,186]. The second component of the CGA entails implementing adjustments to the cancer management plan as needed, based on positive findings. 

The CGA is broadly recommended by an array of professional organizations including the American Society of Clinical Oncology (ASCO), National Comprehensive Cancer Network (NCCN), and International Society of Geriatric Oncology (SIOG) in patients aged 65 or older with cancer. Granular indications vary by organization, but CGA usage is generally recommended in those receiving chemotherapy, and individuals where concerns exist regarding treatment toleration, as well as those with abnormalities identified by a geriatric screening tool [184,185,186]. Common validated instruments include the G8 geriatric screening tool and Vulnerable Elders Survey (VES-13), each of which has been extensively used in thoracic populations and can be completed in under ten minutes. 

How did the CGA become so broadly utilized? These recommendations arise from the demonstrated associations between the CGA and an array of clinically relevant outcomes, including patient and caregiver satisfaction, enhanced communication, quality of life, physical function, decreased treatment toxicity, and possibly survival, although the latter has been less consistently shown [39,40,41,187,188,189,190,191,192,193,194,195,196,197,198,199]. The CGA is also a valuable tool for optimizing non-oncologic care during cancer therapy and increasing advanced care planning [41,197]. Finally, the CGA may be valuable for identifying unmet supportive care needs in older adults, which can subsequently be addressed through the modification of services. For all these reasons, it remains the gold standard of adjunctive assessment for older adults undergoing cancer directed therapy. 

## 4. Age-Specific Considerations for Selection of Lung Cancer-Directed Therapy 

Just as age-specific considerations should inform the provision of medical and supportive care, so too should they shape the selection and delivery of cancer-directed therapy for older adults. In the treatment of lung cancer, appropriate therapy is stage-specific. Thus, for each stage, the following sections outline current treatment guidelines, the ways in which aging may shape their implementation, and recommendations for individualizing and optimizing outcomes of care.

### 4.1. Early-Stage Non-Small Cell Lung Cancer 

For early-stage non-small cell lung cancer (NSCLC), the goal of treatment in the general population is curative, and the mainstay of definitive, local therapy is surgical resection [200]. Eligibility for surgery depends on tumor resectability and medical operability, with the latter largely determined by cardiopulmonary reserve [201]. While cardiac fitness is necessary to withstand general anesthesia, the stresses of surgery, and postoperative recovery, pulmonary reserve is needed to compensate for the loss of resected lung [201]. Thus, patients must undergo pulmonary function testing, ventilation–perfusion imaging, and at times exercise testing to assess suitability [201]. For those deemed medically inoperable or those who decline surgery, definitive radiotherapy (RT), preferably with stereotactic ablative body radiation (SABR), is recommended. Otherwise, image-guided thermal ablation (e.g., cryotherapy, microwave, radiofrequency ablation) remains an option for select patients [200].

In older adults with early-stage NSCLC, management recommendations are notably aligned with general guidelines. Surgery remains the primary treatment of choice, and retrospective studies have shown similar overall survival between elderly and younger patients following resection [202,203]. In one such study, 90-day mortality was comparable among both groups [203]. In another, higher rates of surgery for stages I and II NSCLC were associated with improved one-year survival, even when older and sicker patients underwent resection [204]. Collectively, these data underscore that eligible adults who are medically fit can derive benefits from and should be offered surgical treatment.

Still, important age-related considerations apply. With regard to surgery, normal aging leads to cardiopulmonary changes which can limit physiologic reserve, thereby reducing one’s ability to tolerate perioperative stress [205]. Age-related changes can also predispose individuals to increased comorbidities, in particular cardiovascular pathology and chronic obstructive pulmonary disease (COPD) [205]. In one study, elderly patients were found to be nearly half as likely to undergo resection compared to their younger counterparts; the most frequent reasons for not operating were poor pulmonary function (58%), heart disease (17%), and comorbid illness (17%) [206]. In shaping physiologic changes central to eligibility for therapy, aging can pose particular barriers to the receipt of lung cancer care.

Another necessary consideration involves the tolerance of treatment-related toxicity. For elderly patients with early-stage NSCLC, published results on post-operative morbidity and mortality are currently conflicting, with only some showing a positive association between increasing age and these outcomes [207]. However, for other local therapies, such as radiation, the data may be clearer. Evidence specifically suggests that severe radiation pneumonitis may occur at higher rates in elderly patients compared to younger counterparts [207]. Specific comorbidities, such as collagen vascular diseases, hypersensitivity syndromes, diabetes mellitus, and hypertension, may also negatively impact patients’ ability to tolerate radiotherapy [207]. Given the potential for greater treatment toxicity in older adults, comprehensive assessments, in addition to patient-centered discussions of care goals and values, should be used to inform management decisions.

**Figure 1:**
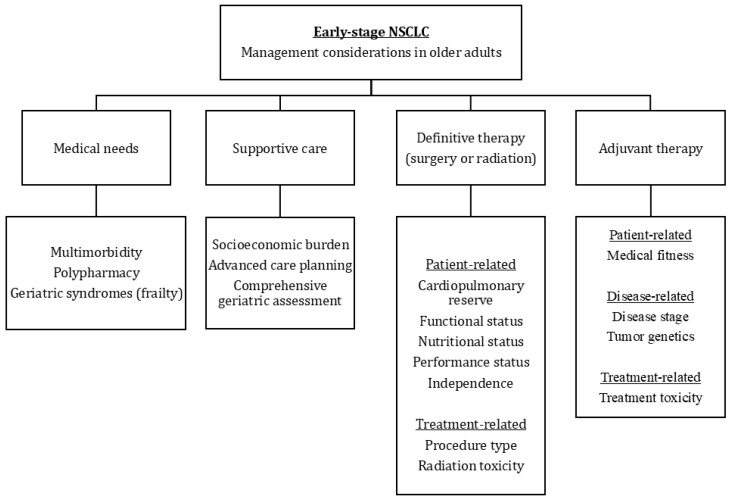
Management considerations in older patients with early-stage non-small cell lung cancer.

Lastly, for those able to receive definitive treatment, special care should be taken during implementation. In the pre-operative setting, in addition to standard evaluations, older adults should undergo optimization of multimorbidity, polypharmacy, functional status, and nutrition (Figure 1) [207]. Clinicians should also consider the use of a number of risk classification systems developed to predict surgical outcomes. Among them, a tool developed by SIOG uses an extended CGA to estimate operative risk, with features such as moderate/severe fatigue, any dependent IADL/ADL, and performance status scores found to be predictive of post-operative complications and extended hospital stay [208]. While planning for surgery, attention should be given to treatment timing and choice of procedure. Broadly, lobectomies are considered standard of care for elderly individuals, and newer surgical approaches such as minimally invasive techniques and video-assisted thoracic surgery may offer the promise of reducing perioperative morbidity or mortality among older adults. In contrast, increased surgery time, extended lung resection, and pneumonectomies have all been associated with poorer outcomes in elderly patients [207]. In tending to these factors, improvements in post-operative outcomes can be attained.

Alongside definitive therapy with either surgery or radiation, a final question in the management of those with early-stage NSCLC revolves around when to offer systemic therapy. Current guidelines recommend that patients of any age with stage IB disease or higher be assessed for preoperative therapy, with strong consideration for chemo-immunotherapy for patients with tumors ≥ 4 cm or with node-positive disease [195]. Additionally, those with disease classified as high-risk stage IB or stages IIA or higher should be indicated for adjuvant therapy [209]. Options for the latter include chemotherapy and radiation, followed by newer immunotherapies and targeted agents [205]. Immune checkpoint inhibitors can be considered for those with stage II EGFR- and ALK- wild-type disease based on PDL-1 expression [210]. Targeted therapies can also be recommended for specific driver alterations (e.g., Osimertinib for EGFR-sensitizing mutations in stage IB/II disease, alectinib for ALK alterations in IB-IIIA disease) [210].

In older adults with early-stage NSCLC, treatment options for systemic therapy are similar, with newer therapies presenting exciting possibilities. As before, adjuvant chemotherapy is still supported for medically fit patients [202]. Concurrently, recent trials with moderate representation of fit older adults have demonstrated the efficacy and tolerability of novel agents, including osimertinib and pembrolizumab, suggesting these options may be viable in some patients [211,212]. Broadly, immunotherapies are considered to be better tolerated compared to chemotherapy, and a recent review on the use of immune checkpoint inhibitors across four major tumor types suggested similar toxicity between older and younger adults [213]. Still, older adults, and frail elderly individuals in particular, remain underrepresented in clinical trials [213]. Thus, decisions around adjuvant therapy should continue to be individualized, taking into account frailty, functional status, and patient preferences. 

### 4.2. Locally Advanced NSCLC 

For locally advanced NSCLC, standard of care consists of multimodal therapy potentially involving surgical resection, local radiation therapy, or systemic therapy (e.g., chemotherapy, immunotherapy, or targeted molecular therapy). If the primary tumor and involved lymph nodes are resectable, guidelines recommend neoadjuvant systemic therapy or concurrent chemoradiotherapy followed by resection; post-surgical treatment includes immunotherapy or targeted molecular therapy if appropriate (e.g., anti-PD-L1 for PD-L1 positive tumors, Osimertinib for EGFR exon 19 deletion or L858R mutation) [200,214]. Post-surgical treatment may also include chemotherapy if not previously offered or radiation in some settings. For inoperable primary tumors, standard treatment is concurrent chemotherapy and radiation therapy followed by systemic consolidation therapy [200,214].

In addition to staging and molecular and genetic testing, treatment considerations include patient performance status, comorbidity burden, cardiac and pulmonary function, systemic therapy tolerability, and socioeconomic supports [200]. In geriatric populations, physicians must also account for multimorbidity, polypharmacy, decreased functional status, and potential geriatric syndromes, particularly frailty [110]. Furthermore, there may be specific concerns related to poor toleration of surgery, immunotherapy, or chemotherapy [215]. Currently, there are limited data addressing the role and feasibility of surgery in elderly patients [215]. For immunotherapy, geriatric patients appear to have similar overall survival outcomes as the general population, but may have an increased risk of developing immune checkpoint inhibitor-related pneumonitis [215]. In terms of chemotherapy, single-agent therapy has been the standard given the risk of increased toxicities with additional agents; although carboplatin-based combinations may provide increased survival benefit, toxicities and treatment-related mortality also appear to increase [215]. Given the lack of data establishing absolute age-specific treatment recommendations for geriatric populations, best practices should include a comprehensive assessment, such as the CGA, to guide treatment decisions appropriate for each patient. 

### 4.3. Metastatic NSCLC

For patients with advanced NSCLC with a known driver mutation, standard of care consists of targeted molecular therapy with chemotherapy; second-line and subsequent treatments may include another targeted agent or combination immunotherapy and chemotherapy [200,216]. For patients without driver mutations, anti-PD-L1 agents alone are recommended for high levels of PD-L1 expression, and combined immunotherapy with chemotherapy is recommended for low levels of PD-L1 expression [200,217].

Similar to other stages of NSCLC, treatment approaches should account for patient performance status, comorbidity burden, and supportive care needs. As described above, physicians must account for additional considerations for geriatric patients, e.g., multimorbidity, polypharmacy, and frailty. For geriatric patients with advanced NSCLC, concerns for poor immunotherapy and chemotherapy toleration are particularly salient [110,218]. Regarding immunotherapy, elderly patients have similar toxicities and overall survival when compared with the general population, however, among patients with ECOG ≥ 2, overall survival is lower [218]. For chemotherapy, patient performance status and frailty should be considered, given fit older adults may benefit from platinum-based combination therapy while their less fit counterparts may be better candidates for monotherapy due to the increased risk of toxicities [218]. Collectively, when caring for geriatric patients with metastatic NSCLC, patients should consider functional status and leverage the CGA to stratify risk and guide treatment decisions. 

## 5. Conclusions and Future Directions

Future work should evaluate the medical and supportive care needs of older adults with NSCLC more granularly across treatment settings. Despite the growing number of elderly patients with NSCLC, there is limited evidence regarding age- and functional status-specific considerations for treatment. For each treatment modality, future studies should further characterize both potential outcome benefits and toxicity risks to better guide clinical decision-making. Furthermore, in the domains of socioeconomic burden and advanced care planning, needs and effective interventions remain under-investigated, impeding potential clinical improvement. Studies evaluating novel interventions in older adults must consider outcomes beyond overall survival, including quality-of-life, symptom burden, and functional outcomes.

Future research should also prioritize the integration of geriatric assessment tools into clinical trials for older patients with NSCLC. By comprehensively evaluating frailty, comorbidities, functional status, and cognitive impairment, these tools may be able to identify appropriate patients for treatment intensification and deintensification. Developing and validating treatment algorithms grounded in geriatric assessment will be crucial for supporting evidence-based decision-making appropriate for each patient. Coupling these algorithms with age-specific biomarkers and molecular profiling could further enhance treatment personalization, improving outcomes.

Across disease stages, older adults with NSCLC have a varied array of medical, psychosocial, and supportive care needs. Enhancing care in this population requires a multifaceted approach integrating comprehensive evaluation with personalized treatment planning. By further delineating patient needs and evaluating geriatric assessment tools across settings, future work has the potential to enhance treatment efficacy, reduce age-based disparities in care delivery, and minimize unnecessary symptom burden. Ultimately, care should be individualized, informed by evidence-based evaluation, and aligned with each patient’s overall health, functional status, and care goals.

## Figures and Tables

**Table 1:** Brief assessment tools for domains related to symptom burden and experiences of older patients with non-small cell lung cancers.

Domain	Examples of Brief Assessment Tools
Medical: Multimorbidity	Charlson Comorbidity Index (CCI) [43,44,45,46] Adult Comorbidity Evaluation-27 (ACE-27) [47] Cumulative Illness Rating Scale-Geriatric (CIRS-G) [48] Mini-COG [49]
Medical: Polypharmacy/PIMs	American Geriatrics Society Beers Criteria [50] STOPP/START Criteria [51,52]
Medical: Frailty and Geriatric Syndromes	Pictoral Fit-Frail Scale (PFFS) [53,54] Carolina Frailty Index (CFI) [55] Balducci Classification [56] SIOG 1/2 Classification [56]
Supportive Care: Treatment-Related Symptom Burden, QOL, and Functional Status	Patient Health Questionnaire for Anxiety and Depression (PHQ-4) [57] Geriatric Depression Scale (GDS-15) [58] Revised Edmonton Symptom Assessment System (ESAS-r) [59] MDASI-LC [60] EORTC-QLQ-LC13 [61] EORTC QLQ-C30 [62] EORTC QLQ-ELD-14 [63] VES-13 [64]
Supportive Care: Socioeconomic Supports & Burdens	Comprehensive Score for Financial Toxicity (COST) [65,66] Caregiver Reaction Assessment (CRA) [67] CareGiver Oncology Quality of Life Questionnaire (CarGOQoL) [68] Zarit Caregiver Burden Interview (ZBI) [69,70] 8-Item Medical Outcomes Study Social Support Survey [71]
Supportive Care: Advanced Care Planning	Respecting Choices [72,73,74,75,76] Prepare for Your Care [72] VIDEO-PCE [73]

Note that multiple screening tools for global geriatric vulnerability meriting an expedited CGA also exist, including the VES-13 (which also measures functional status), and the G8 [77,78]. We confirm that this table is original work and has not been previously published.
